# Severe Hypothyroidism Presenting as Rhabdomyolysis and Acute Kidney Injury: A Report of a Rare Case

**DOI:** 10.7759/cureus.112029

**Published:** 2026-07-04

**Authors:** Kawtar Dadi, Ali Halouache, Lahoussaine Abainou, Marouane Jabrane, Mohamed Arrayhani, Mounia Azizi, Ahmed Alaayoud

**Affiliations:** 1 Nephrology and Kidney Transplantation, Mohammed VI University Hospital Center, Agadir, MAR; 2 Endocrinology, Oued Eddahab Military Hospital, Agadir, MAR; 3 Nephrology, Oued Eddahab Military Hospital, Agadir, MAR

**Keywords:** acute kidney injury, acute tubular necrosis, hashimoto’s thyroiditis, hypothyroidism, rhabdomyolysis

## Abstract

Hypothyroidism is a rare cause of rhabdomyolysis and an even less common cause of acute kidney injury (AKI). Because the symptoms may be mild or non-specific, the diagnosis can easily be overlooked. We report the case of a 44-year-old man with no significant past medical history who was admitted after renal impairment was discovered on routine blood testing. On clinical examination, he had sinus bradycardia, puffy facies with bilateral periorbital edema, Hertoghe sign, muffled heart sounds, psychomotor slowing, and constipation. Laboratory investigations showed stage II AKI, severe rhabdomyolysis with creatine phosphokinase levels above 8,450 IU/L (reference range: <171 IU/L), and liver cytolysis. Further evaluation revealed profound hypothyroidism, with markedly elevated thyroid-stimulating hormone, very low free thyroxine, and strongly positive anti-thyroid peroxidase antibodies, consistent with autoimmune thyroiditis. Thyroid ultrasound supported the diagnosis of Hashimoto’s thyroiditis, and echocardiography showed a moderate pericardial effusion without hemodynamic compromise. Because nephrotic-range proteinuria was also present, a kidney biopsy was performed and showed mainly tubulointerstitial injury with pigmented casts and tubular atrophy, associated with focal segmental glomerulosclerosis. The patient was treated with intravenous hydration and gradual levothyroxine replacement, with clear clinical improvement and progressive normalization of laboratory abnormalities. This case highlights that severe hypothyroidism may rarely present with rhabdomyolysis and AKI. It also underlines the importance of considering thyroid dysfunction in patients with unexplained rhabdomyolysis, especially when the clinical picture is not very suggestive, since early treatment can lead to a favorable outcome.

## Introduction

Acute kidney injury (AKI) is a common complication in clinical practice, with various causes. Among them is rhabdomyolysis, which results from the destruction of muscle cells releasing myoglobin, leading to kidney injury through toxic acute tubular necrosis. Non-traumatic causes of rhabdomyolysis include metabolic disorders, drug intoxication, severe infections, and, more rarely, endocrine disorders such as hypothyroidism.

The coexistence of hypothyroidism, rhabdomyolysis, and AKI remains exceptional, with only a few cases reported in the literature [1,2]. The diagnosis is often delayed because the clinical presentation is not very obvious, which can compromise renal prognosis. This case highlights the importance of screening for hypothyroidism in patients presenting with unexplained rhabdomyolysis complicated by AKI.

## Case presentation

We report the case of a 44-year-old man with no significant medical history, no chronic medication use, and no history of alcohol consumption or illicit drug use, admitted to our unit for investigation of renal impairment discovered during routine testing. He also denied recent trauma or strenuous physical activity.

On admission, physical examination showed sinus bradycardia (54 bpm) with stable blood pressure (134/76 mmHg). He had a puffy facial appearance with bilateral periorbital edema and a positive Hertoghe sign (characterized by thinning of the lateral third of the eyebrows), along with muffled heart sounds and clear psychomotor slowing. He also reported a tendency to constipation, without profound asthenia or other associated symptoms.

The initial laboratory evaluation (Table 1) confirmed Acute Kidney Injury Network (AKIN) stage II AKI associated with severe rhabdomyolysis, hepatic cytolysis, and profound primary hypothyroidism. No history of trauma or strenuous physical activity was reported. Blood urea nitrogen, serum potassium, and phosphate levels were within normal ranges, excluding hypokalemia and hypophosphatemia as alternative metabolic causes of rhabdomyolysis. Plasma cortisol levels were also normal, ruling out associated adrenal insufficiency.

**Table 1:** Initial laboratory findings. UPCR: urine protein-creatinine ratio

Parameter	Result	Reference range
Renal parameters		
Serum creatinine	26 mg/L	7-12 mg/L
Blood urea nitrogen (BUN)	0.73 g/L	0.15-0.45 g/L
Proteinuria (UPCR)	4 g/g	<0.15 g/g
Albuminuria	1.4 g/L (≈2.1 g/24 h)	<30 mg/24 h
Hematuria	<10,000/mL	Negative
Uric acid	120 mg/L	35-70 mg/L
Muscle injury and liver enzymes		
Creatine kinase (CK)	>8,450 IU/L	<171 IU/L
Lactate dehydrogenase (LDH)	1,055 IU/L	125-220 IU/L
Aspartate aminotransferase (AST)	153 IU/L	<40 IU/L
Alanine aminotransferase (ALT)	33 IU/L	<40 IU/L
Electrolytes and acid-base status		
Sodium	140 mmol/L	136-145 mmol/L
Potassium	4.3 mmol/L	3.5-5.1 mmol/L
Phosphate	32 mg/L	25-45 mg/L
Calcium	94 mg/L	88-102 mg/L
Bicarbonate (HCO₃⁻)	18 mmol/L	22-29 mmol/L
Hematological parameters		
Hemoglobin	11.2 g/dL	13-17 g/dL
White blood cell count	4.5 × 10⁹/L	4-10 × 10⁹/L
Platelet count	241 × 10⁹/L	150-400 × 10⁹/L
Thyroid and endocrine evaluation		
Thyroid-stimulating hormone (TSH)	>99.99 µIU/mL	0.35-4.94 µIU/mL
Free thyroxine (FT4)	0.43 ng/dL	0.70-1.48 ng/dL
Anti-thyroid peroxidase antibodies (anti-TPO)	>1,000 IU/mL	<5.6 IU/mL
Morning cortisol (8 AM)	177 nmol/L	101.2-535.7 nmol/L
Inflammatory and nutritional parameters		
C-reactive protein (CRP)	0.23 mg/dL	<0.5 mg/dL
Serum albumin	38 g/L	35-50 g/L

**Figure 1:**
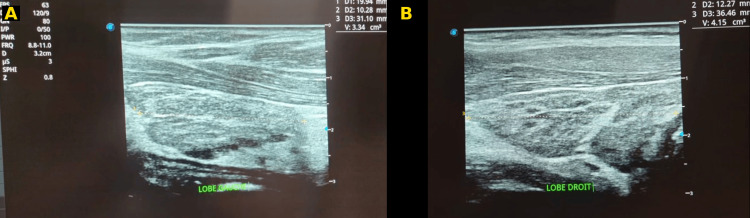
Thyroid ultrasonography showing bilateral gland atrophy with heterogeneous echotexture. Gray-scale ultrasound images showing reduced thyroid volume and diffuse heterogeneous echotexture involving both lobes. (A) Left thyroid lobe (volume 3.34 cm³). (B) Right thyroid lobe (volume 4.15 cm³).

Further investigations supported the diagnosis of Hashimoto’s thyroiditis. Cervical ultrasound showed a reduced thyroid gland with a heterogeneous echotexture and loss of the musculo-parenchymal gradient, findings consistent with chronic autoimmune thyroiditis. Transthoracic echocardiography revealed a moderate pericardial effusion without hemodynamic compromise. Electromyography was normal, ruling out an inflammatory or neurogenic myopathy and supporting a metabolic etiology for the rhabdomyolysis (Figure 1).

**Figure 2:**
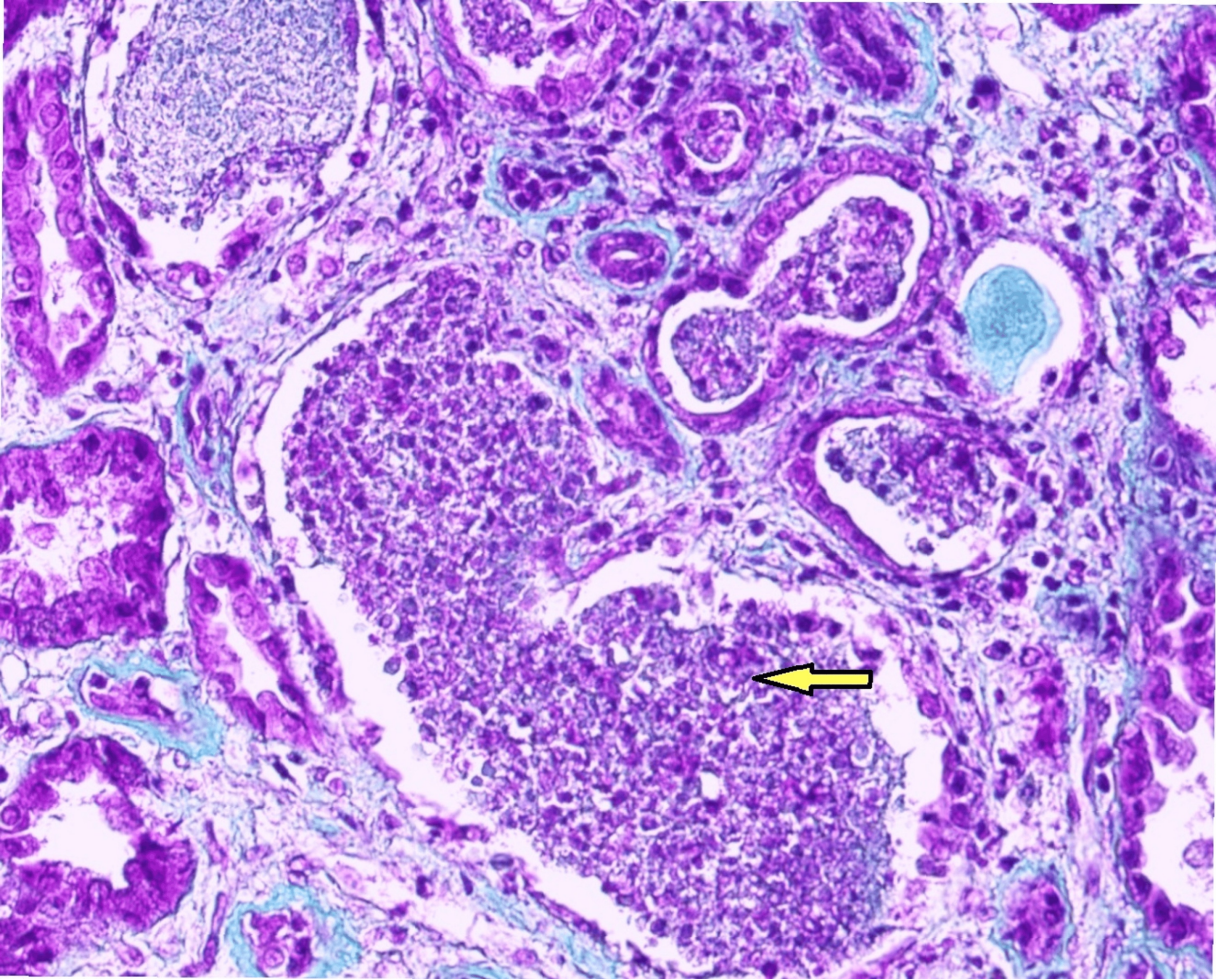
Kidney biopsy demonstrating pigmented tubular casts. Histopathological examination of the kidney biopsy showing intratubular pigmented casts (arrow) and associated tubulointerstitial injury, consistent with rhabdomyolysis-associated acute kidney injury (Masson's trichrome stain).

However, the patient also had nephrotic-range proteinuria (urine protein-creatinine ratio (UPCR) 4 g/g), associated with significant albuminuria (1.4 g/L, approximately 2.1 g/24 h), raising the possibility of an underlying glomerular lesion and prompting a kidney biopsy. Histological examination mainly revealed tubulointerstitial injury characterized by granular pigmented casts and tubular atrophy, with associated focal segmental glomerulosclerosis (FSGS) lesions (Figure 2).

Treatment consisted of gradual thyroid hormone replacement with oral levothyroxine, initiated at 12.5 µg/day and increased by 12.5 µg every five days under close clinical monitoring, including regular assessment of vital signs and electrocardiographic surveillance. In view of the severe hypothyroidism and pending endocrine evaluation, hydrocortisone hemisuccinate was administered at a dose of 15 mg in the morning and 5 mg at 4 PM. In addition, the patient received intravenous isotonic saline hydration (3 L/day) according to the institutional rhabdomyolysis protocol. No hypotension was observed during hospitalization. The patient's clinical course was favorable, with marked clinical improvement and significant regression of biological abnormalities.

## Discussion

To date, the association between severe hypothyroidism, rhabdomyolysis, and AKI remains rare [3]. Although hypothyroid myopathy is common, progression to severe rhabdomyolysis complicated by AKI has rarely been reported in the literature.

The pathophysiology of rhabdomyolysis is related either to direct injury to the sarcolemma (through direct trauma) or to adenosine triphosphate (ATP) depletion within myocytes, leading to uncontrolled leakage of extracellular calcium into the intracellular compartment [4]. Sarcoplasmic calcium is tightly regulated by a series of energy-dependent ion pumps, such as Na^+^/K^+^ ATPase and Ca^2+^ ATPase in the sarcolemma, as well as in other intracellular membranes. These pumps maintain low intracellular calcium concentrations when the muscle is at rest and high concentrations when muscle contraction is required. Regardless of the underlying mechanism, muscle injury results in increased sarcoplasmic calcium and sustained contraction, leading to necrosis of muscle fibers and massive release of muscle enzymes and electrolytes into the extracellular and intravascular spaces [5].

The massive release of myoglobin secondary to this necrotic phenomenon exposes patients to a risk of AKI. Myoglobin, which is a protein, freely filtered by the glomerular basement membrane and then metabolized by tubular cells, accumulates in the latter when the serum concentration exceeds the renal clearance level [6]. It becomes concentrated within the renal tubules, a process worsened by dehydration and renal vasoconstriction. Its precipitation with Tamm-Horsfall protein is facilitated by urinary acidity, resulting predominantly in distal tubular obstruction [7], whereas direct tubular cytotoxicity is mainly observed in the proximal tubules. Moreover, intrarenal vasoconstriction associated with rhabdomyolysis exacerbates tubular ischemia [8]. The cumulative effect of these pathways is a decline in glomerular filtration rate. This mechanism was reflected in our patient’s renal biopsy, which demonstrated tubulointerstitial lesions and obstructive pigmented casts.

An additional finding on the kidney biopsy was the presence of FSGS lesions. These lesions may account for the patient's nephrotic-range proteinuria. However, the predominant histological findings consisted of pigmented casts and tubulointerstitial injury, supporting rhabdomyolysis-associated AKI as the main mechanism underlying the acute presentation. As hypothyroidism is not a recognized cause of FSGS and no previous renal evaluation was available, the exact origin and chronicity of these glomerular lesions could not be determined. Therefore, the FSGS was considered an associated glomerular finding rather than the primary driver of the AKI.

Hypothyroidism represents a rare non-traumatic etiology of rhabdomyolysis, and the exact mechanisms are not fully elucidated. A deficiency in thyroid hormones disrupting glycogenolysis and mitochondrial oxidative metabolism has been proposed as an explanatory mechanism [9].

Although rhabdomyolysis classically presents with myalgia, muscle weakness, and cola-colored urine secondary to myoglobinuria, many patients do not exhibit the complete clinical triad. Therefore, the absence of these manifestations should not preclude consideration of severe hypothyroidism as an underlying cause of rhabdomyolysis [3]. Indeed, several published observations describe cases of rhabdomyolysis occurring without any discernible triggering factor, including absence of exertional stress, absence of implicated medications (statins, antibiotics, etc.), and no concomitant infectious syndrome [10,11]. This is in keeping with the presentation observed in our patient.

Despite markedly elevated creatine kinase levels, the clinical presentation was dominated by features of severe hypothyroidism rather than manifestations directly attributable to rhabdomyolysis. This clinicobiological dissociation may contribute to diagnostic delay and highlights the importance of considering severe hypothyroidism in cases of unexplained rhabdomyolysis.

In rare situations, rhabdomyolysis may reveal undiagnosed hypothyroidism or occur in patients with poor treatment adherence [11,12]. In these cases, diagnosis should be established promptly, with a high level of suspicion, in order to prevent complications of rhabdomyolysis, notably AKI and severe metabolic disorders potentially requiring hemodialysis or admission to an intensive care unit [13].

Therapeutic management is essentially based on volume expansion and progressive correction of hypothyroidism. The favorable outcome observed in our patient, characterized by improvement in rhabdomyolysis and renal function, emphasizes the importance of optimal replacement therapy.

## Conclusions

This case underscores a rare association between profound hypothyroidism, rhabdomyolysis, and AKI occurring in the absence of an identifiable triggering factor. The lack of specific clinical manifestations may lead to delayed diagnosis, whereas early management is determinant for outcome. Consequently, systematic assessment of thyroid function in unexplained rhabdomyolysis appears justified, particularly as thyroid hormone replacement is generally associated with rapid improvement in biological abnormalities and renal function.
